# RASAL2 down-regulation in ovarian cancer promotes epithelial-mesenchymal transition and metastasis

**DOI:** 10.18632/oncotarget.2244

**Published:** 2014-07-23

**Authors:** Yuting Huang, Meng Zhao, Haixu Xu, Ke Wang, Zheng Fu, Yuan Jiang, Zhi Yao

**Affiliations:** ^1^ Tianjin Medical University Cancer Institute and Hospital, National Clinical Research Center of Cancer, Key Laboratory of Cancer Prevention and Therapy, Tianjin, P.R. China; ^2^ Department of Immunology, Tianjin Key Laboratory of Cellular and Molecular Immunology, Key Laboratory of Educational Ministry of China, School of Basic Medical Sciences, Tianjin Medical University, Tianjin, P.R. China

**Keywords:** ovarian cancer, RASAL2, EMT, MAPK, metastasis

## Abstract

Ovarian cancer is the most lethal gynecologic malignancy, and transcoelomic metastasis is responsible for the greatest disease mortality. Although intensive efforts have been made, the mechanism behind this process remains unclear. RASAL2 is a GTPase activating proteins (GAPs) which was recently reported as a tumor suppressor in breast cancer. In this study, we identified RASAL2 as a regulator of epithelial-mesenchymal transition (EMT) and metastasis in ovarian cancer. RASAL2 was down-regulated in ovarian cancer samples compared with normal tissue samples, especially in advanced stages and grades. RASAL2 knockdown in ovarian cancer cell lines promoted *in vitro* anchorage-independent growth, cell migration and invasion and *in vivo* tumor formation. Moreover, we observed EMT in RASAL2-depleted cells. E-cadherin-mediated cell-cell adhesion was attenuated, and mesenchymal markers were up-regulated. Further investigation revealed that the oncogenic role of RASAL2 down-regulation was mediated by the Ras-ERK pathway. RASAL2 knockdown activated the Ras-ERK pathway, and inhibition of the pathway reversed the functional effects of RASAL2 depletion. Together, our results implicate RASAL2 as an EMT regulator and tumor suppressor in ovarian cancer, and down-regulation of RASAL2 promotes ovarian cancer progression.

## INTRODUCTION

Ovarian cancer represents the most lethal gynecological malignancy and ranks fifth in female cancer-related deaths. In 2014, the estimated new cases and deaths due to ovarian cancer in the United States were 21,980 and 14,270, respectively. The incidence of ovarian cancer accounts for up to 3% of female malignant diseases, while the mortality accounts for up to 5%[1]. The symptoms for early diagnosis are difficult to detect; consequently, more than 70% of patients are already in the advanced stages of disease by the time they are diagnosed (FIGO III-IV) [2]. Surgical management remains difficult and inefficient because metastasis has already occurred when the treatment is given. The five-year survival rate of patients in advanced stages fluctuates at approximately 40%[1]. Exploration of mechanisms of ovarian cancer metastasis has become a prerequisite for improving ovarian cancer therapeutics.

Unlike other malignant tumors, ovarian cancer can directly invade adjacent tissues and spread in ascites within the peritoneal cavity. Epithelial-mesenchymal-transition (EMT) plays a key role in the metastasis of ovarian cancer[3-6].

EMT is characterized by a phenotypic and functional alteration in epithelial cells with inherent polarity that grow on the basement membrane. The transition includes the loss of epithelial features, such as cellular polarization, in conjunction with the acquisition of mesenchymal properties, such as invasive and migratory capacity, as well as other biological abilities, such as escape from apoptosis and secretion of extracellular matrix (ECM) [7-10]. EMT is a physiological mechanism that participates in embryogenesis, organ development, wound healing and repair. Under pathological conditions, EMT contributes to organ fibrosis; moreover, it is closely associated with tumor metastasis[7]. Tumor cells may gain the ability to invade and migrate using pathways that EMT utilizes during embryogenesis and wound healing. Recent studies have also suggested a link between the EMT phenotype and the stemness of tumor cells[11, 12].

The Ras pathway, one of the most studied pathways in cancer, is highly prone to abnormality. *RAS* mutations are very common, being found in a broad spectrum of malignant diseases[13]. However, the inappropriate activation of Ras pathway is not only due to *RAS* mutations; rather, changes in its upstream and downstream regulatory factors can also alter the activation of the Ras pathway. Activation and deactivation of Ras family members are antagonistic regulated by guanine nucleotide exchange factors (GEFs) and GTPase activating proteins (GAPs), respectively[14, 15]. Compared with the large number of reports on *Ras* mutations, research on the impact of post-translational modifications of *Ras* during ovarian cancer is rarely reported.

RASAL2, encoded by the *RASAL2* gene, is a RasGAP. Weeks et al. identified RASAL2 as a mesenchymal-amoeboid transition regulator in human astrocytoma cells[16]. Interestingly, recent studies uncovered that it has a tumor suppressor role in breast cancer[17]. In the present study, we explored RASAL2′s potential role in ovarian cancer. We found that the expression of RASAL2 is down-regulated in ovarian cancer, and this down-regulation is related to clinical features. Following RASAL2 depletion, ovarian cancer cells showed enhanced invasion and migration, anchorage-independent growth and tumor formation. Moreover, EMT was also observed, which was characterized as increased expression of epithelial-related markers and decreased expression of mesenchymal-related markers. Finally, these changes were primarily mediated via the Ras-ERK pathway.

## RESULTS

### RASAL2 is down-regulated in ovarian cancer tissue and correlates with pathological grade and FIGO stage

**Figure 1:**
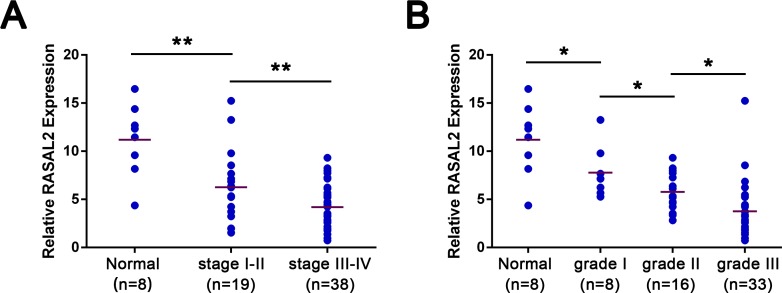
Expression of RASAL2 in ovarian cancer specimens cDNA from 57 patients representing all disease stages and grades and 8 normal ovarian epithelium samples were analyzed for RASAL2 levels by qRT-PCR. (A) Relative levels of RASAL2 mRNA in normal ovaries or stage I-IV ovarian cancer. (B) Relative levels of RASAL2 mRNA in normal ovaries or grade 1-3 ovarian cancer. Bars, mean per group. *, P < 0.05, * *, P < 0.01. Details of patient samples are described in Supplementary Table S1.

To delineate the extent of RASAL2 expression alterations in ovarian cancer, we analyzed RASAL2 expression by qRT-PCR in cDNA from 57 patients representing all stages and grades of the disease and 8 normal ovarian epithelium samples (Table 1). The relative expression of RASAL2 mRNA was normalized to glyceraldehyde-3-phosphate dehydrogenase (GAPDH) mRNA. The results showed that RASAL2 expression was signiﬁcantly lower in tumor samples than in normal ovarian tissue samples, which are postulated to be the tissues of origin for the majority of ovarian carcinomas (Figure 1).

**Table 1:** Distribution by tumor characteristics for ovarian cancer patients

Feature	
Mean Age at diagnosis (y)	54.87
Histotype (n)	
Serous	30
Endometrioid	14
Mucinous	9
Clear cell	4
Stage (n)	
I	4
II	15
III	10
IV	18
Grade (n)	
1	8
2	16
3	33
Total (n)	57

Furthermore, statistical analysis showed that RASAL2 expression was not signiﬁcantly correlated with age or histology type. However, its expression level was signiﬁcantly higher in ovarian carcinomas with FIGO stage I–II than with stage III–IV (Figure 1A). Notably, decreased levels of RASAL2 mRNA were observed with increasing pathological grades of the disease (Figure 1B). Taken together, these findings show that RASAL2 is down-regulated in primary ovarian cancers, and this decreased expression is correlated with pathological grade and FIGO stage. These data suggest that RASAL2 may play a role as a tumor suppressor in ovarian cancer.

### RASAL2 regulates ovarian cancer cell invasion and anchorage-independent growth *in vitro* and tumor formation *in vivo*

**Figure 2:**
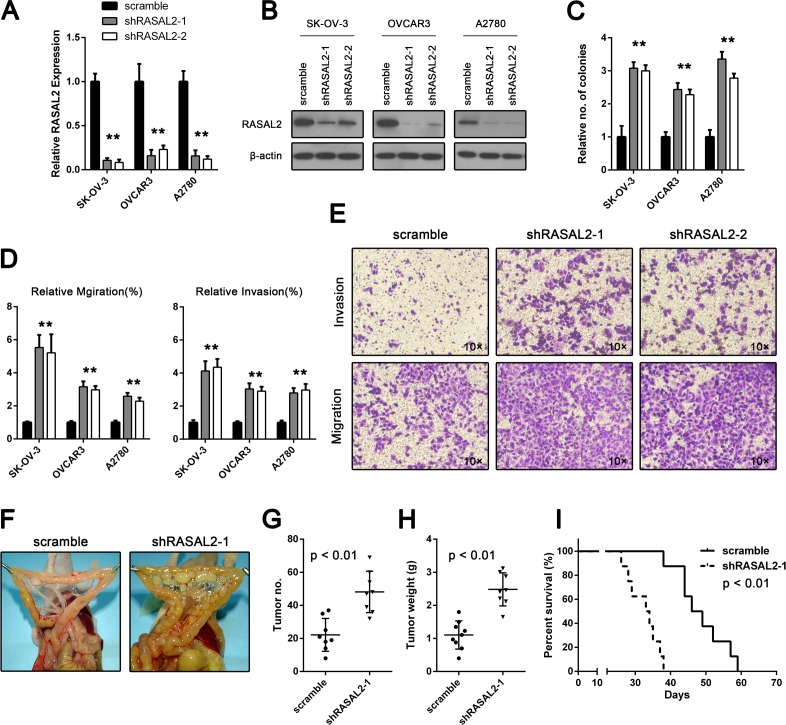
RASAL2 regulates *in vitro* ovarian cancer cell invasion and anchorage-independent growth and *in vivo* tumor formation (A and B) qPCR and Western blot analysis of RASAL2 levels in SK-OV-3, OVCAR3 and A2780 cells infected with shRASAL2-1, shRASAL2-2 or scramble-shRNA. Shown are the average results of 3 independent experiments ± SD. (C) Anchorage-independent growth of SK-OV-3, OVCAR3 and A2780 cells expressing scramble or shRASAL2-1 or -2 shRNA. The graph indicates relative number of colonies ± SD (n = 3). (D) Assays for cell migration (left) or invasion (right) on SK-OV-3, OVCAR3 and A2780 cells infected with shRASAL2-1, shRASAL2-2 or scramble-shRNA. All the data were normalized to the results of cells transfected with scramble-shRNA. The data are shown as the means ± SD (n = 3). (E) Representative graphs of SK-OV-3 cells in migration or invasion assays. (F, G and H) Female nude mice were injected i.p. with 2×10^6^ SK-OV-3 cells infected with either shRASAL2-1 or scramble-shRNA. Three weeks after injection, the mice were euthanized. Tumor number and weight were measured. Bars, mean of each group ± SD (n = 8). (I) Kaplan-Meier survival curves of mice injected i.p. with 2×10^6^ SK-OV-3 cells infected with either shRASAL2-1 or scramble-shRNA. *, P < 0.05, * *, P < 0.01.

To functionally validate the role of RASAL2 in ovarian cancers, we suppressed the expression of RASAL2 in ovarian cancer cell lines (SK-OV-3, OVCAR3 and A2780) using short-hairpin RNAs (shRNA), which targeted 2 different sequences in RASAL2. The expression of RASAL2 mRNA and protein were inhibited in cells transfected with either RASAL2-shRNA1 or RASAL2-shRNA2 (shRASAL2-1 or shRASAL2-2) in contrast to cells transfected with a control shRNA (scramble; Figure 2A and 2B). Proliferation and apoptosis were unaffected (Supplementary Figure 1); however, RASAL2 knockdown cells produced significantly more colonies than control cells in an anchorage-independent growth assay (Figure 2C).

**Figure 3:**
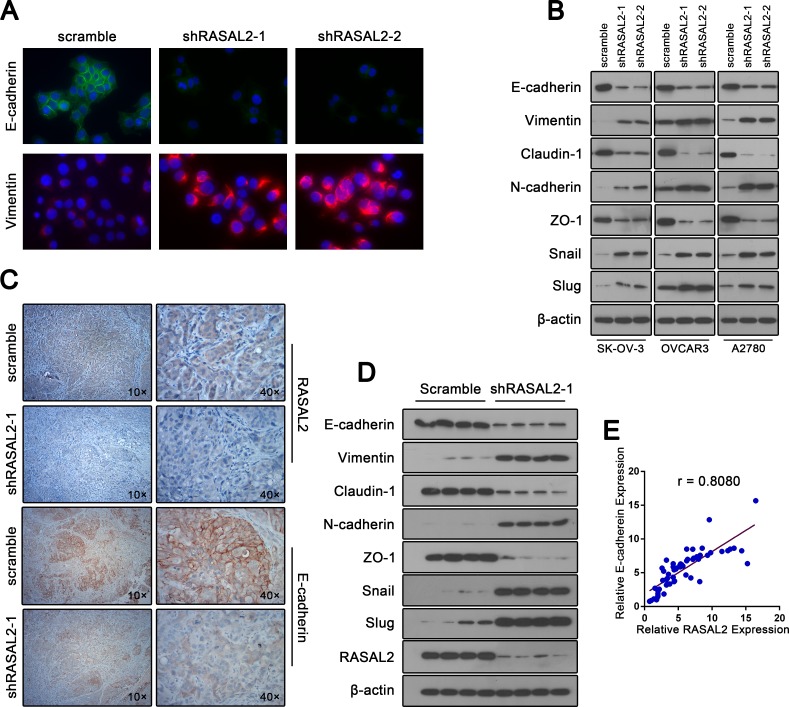
RASAL2 regulates EMT in ovarian cancer (A) Expression of E-cadherin or vimentin was detected by immunofluorescence staining in SK-OV-3 cells infected with shRASAL2-1, shRASAL2-2 or scramble-shRNA under 40x magnification. (B) Western blot analysis of EMT markers in SK-OV-3, OVCAR3 and A2780 cells expressing scramble or shRASAL2-1 or -2 shRNA. (C) Xenograft tumor sections (SK-OV-3) were stained with either anti-RASAL2 antibody or anti-E-cadherin antibody. (D) Western blot analysis of EMT marker alterations in protein lysates from xenograft SK-OV-3 tumors. (E) Pearson correlation analysis on RASAL2 and E-cadherin. The relative expression of RASAL2 and E-cadherin in 57 patients ovarian cancer and 8 normal ovarian epithelium samples was determined by qPCR. Then, the correlation between RASAL2 and E-cadherin expression was examined. Details of patient samples are described in Supplementary Table S1.

Migration and invasion are critical steps in the initial progression of cancer and facilitate metastasis[18]. To further investigate the signiﬁcance of RASAL2 down-regulation in ovarian cancer, we determined whether RASAL2 suppression might also promote migration and invasion. Migration was assessed using the short-term transwell migration assay. RASAL2 suppression signiﬁcantly increased the migration of SK-OV-3, OVCAR3 and A2780 cells compared with the control cell lines (Figure 2D and 2E). We further confirmed these findings by using a wound-healing assay. In agreement with the results obtained from the transwell migration assay, RASAL2 suppression increased the migration of SK-OV-3, OVCAR3 and A2780 cells (Supplementary Figure 2). To assess cell invasiveness, we used Matrigel-coated Boyden chambers. Invasion by SK-OV-3, OVCAR3, and A2780 cells was significantly increased by RASAL2 knockdown (Figure 2D and 2E). The increased invasiveness and migration by RASAL2 depletion was not due to the altered viability of cells because cell proliferation and apoptosis were not affected by RASAL2 suppression (Supplementary Figure 1).

Unlike other malignant epithelial diseases, epithelial ovarian cancer most frequently disseminates via the transcoelomic route[19]. To further verify the oncogenic role of RASAL2 knockdown, we established ovarian carcinoma orthotopic mouse models using the SK-OV-3 cell line to mimic orthotopic implantation metastases. SK-OV-3 cells treated with shRASAL2-1 or scramble shRNA were injected into the peritoneal cavities of female athymic nude mice. As shown in Figure 2F, RASAL2 suppression resulted in a signiﬁcant increase in the number of tumor nodules (Figures 2G) and tumor weight (Figure 2H) compared with scramble. Furthermore, knockdown of RASAL2 significantly shortened the survival of tumor-bearing mice (Figure 2I). Taken together, these results confirm the oncogenic role of RASAL2 down-regulation in ovarian cancer, especially its effects on invasion and migration.

### RASAL2 regulates EMT in ovarian cancer

EMT is associated with malignant properties, such as invasion and anchorage-independent growth[9]. Several biochemical markers are used to characterize EMT: epithelial cells predominantly express E-cadherin, claudin-1 and ZO-1, whereas mesenchymal cells express vimentin, and N-cadherin[20, 21]. Because the down-regulation of RASAL2 promotes migration and invasion, we determined whether RASAL2 suppression was sufficient to induce EMT by examining expression of the aforementioned markers.

Suppressing RASAL2 with either RASAL2-shRNA1 or RASAL2-shRNA2 in either SK-OV-3, OVCAR3 or A2780 cells attenuated the mRNA expression of the epithelial markers E-cadherin, claudin-1 and ZO-1 but augmented the mRNA expression of the mesenchymal markers vimentin and N-cadherin (Supplemental Figure 3). Consistent with the qPCR results, the protein profile of EMT markers switched from epithelial to mesenchymal (Figure 2B). Moreover, immunofluorescence microscopy confirmed increased levels of E-cadherin and decreased levels of vimentin in RASAL2 knockdown SK-OV-3 cells compared with control cells (Figure 2A).

The transcription factors Snail and Slug have been implicated in the transcriptional repression of E-cadherin and in orchestrating the molecular EMT program. Because RASAL2 knockdown suppressed E-cadherin and increased vimentin expression, we determined whether RASAL2 regulated these transcription factors. In agreement with the above results, both qRT-PCR and Western blot analysis demonstrated clear up-regulation of Snail and Slug in RASAL2 knockdown cells (Supplemental Figure 3 and Figure 2B).

We further investigated whether RASAL2 depletion promoted EMT of ovarian cancer cells *in vivo*. Analyses of xenograft tumor sections revealed a loss of the epithelial nature of the tumor following RASAL2 knockdown in SK-OV-3 cells as evidenced by decreased E-cadherin expression (Figure 2C). Western blot analysis of protein extracted from xenograft tumors confirmed the switch in EMT markers due to RASAL2 knockdown (Figure 2D).

Finally, we examined whether RASAL2 expression correlated with E-cadherin expression in ovarian cancer patient samples. The mRNA expression levels of RASAL2 and E-cadherin were evaluated in 57 patients ovarian cancer and 8 normal ovarian epithelium samples by quantitative RT-PCR. As expected, a signiﬁcant correlation between RASAL2 and E-cadherin expression was observed (r = 0.808; Figure 2E). Taken together, these results indicate that RASAL2 is crucial for ovarian cancer to maintain epithelial characteristics.

### RASAL2 knockdown activates the Ras-ERK pathway

**Figure 4:**
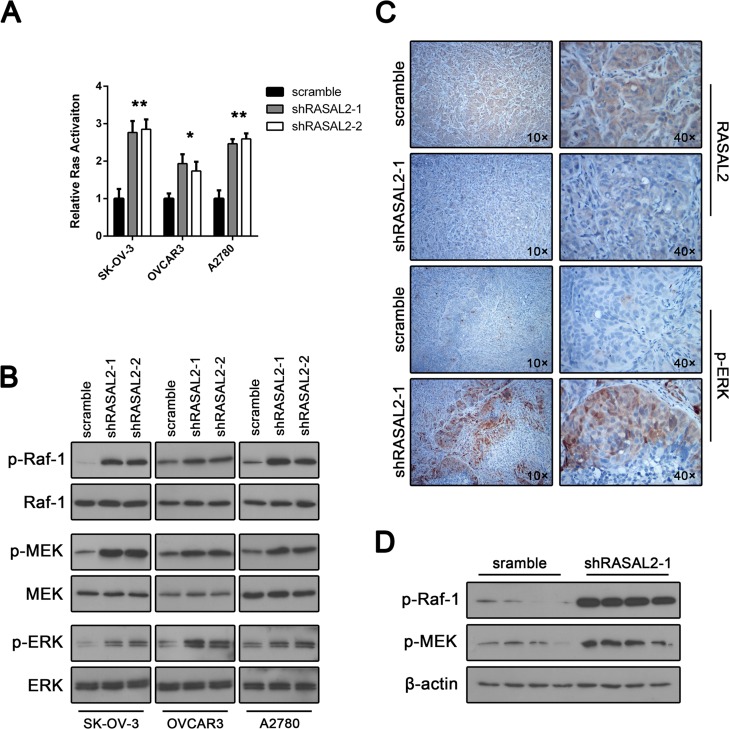
RASAL2 knockdown activates the Ras-ERK pathway (A) The effect of RASAL2 knockdown on Ras activation in SK-OV-3, OVCAR3 and A2780 cells was evaluated by a Ras Activation ELISA Assay Kit. All the data were normalized to the results of cells transfected with scramble-shRNA. The data are shown as the means ± SD (n = 3). (B) Phosphorylation of Raf-1, MEK1/2 and ERK1/2 in SK-OV-3, OVCAR3 and A2780 cells infected with shRASAL2-1, shRASAL2-2 or scramble-shRNA was analyzed by Western blot. Xenograft tumor sections (SK-OV-3) were also examined for levels of p-ERK by IHC (C) and for p-Raf-1 and p-MEK by Western blot (D).

Because RASAL2 belongs to the RasGAP family, we investigated whether RASAL2 regulated the Ras-ERK pathway in ovarian cancer. Initially, we asked whether RASAL2 knockdown could alter Ras activation by measuring levels of Ras-GTP. Compared with control cells, RASAL2 suppression led to a clear up-regulation of Ras-GTP in SK-OV-3, OVCAR3 and A2780 cells (Figure 4A). Because Raf-1 is phosphorylated and activated by Ras proteins, we next investigated the phosphorylation status of Raf-1 at regulatory serines in their activation loops. Although the total levels of Raf-1 remained unchanged, ovarian cancer cells transfected with either RASAL2-shRNA1 or RASAL2-shRNA2 showed a clear induction of Raf-1 phosphorylation compared with control cells (Figure 4B).

MEK1 and MEK2 are activated by phosphorylation catalyzed by Raf family kinases. We examined their activation status by using an anti-phospho antibody. Again, phospho-MEK1/2 displayed clear up-regulation in ovarian cancer cell lines after RASAL2 depletion (Figure 4B). ERK1 and ERK2 are the core effectors of the ERK pathway. RASAL2 knockdown led to a strong increase in the phosphorylation of ERK1 and ERK2 in SK-OV-3, OVCAR3 and A2780 cells, whereas Western blot analysis showed equivalent levels of total ERK1/2 (Figure 4B).

We determined whether the ERK pathway was also activated in a xenograft tumor model. Immunohistochemical analyses were conducted to detect ERK1 and ERK2 activation status by staining for anti-p-ERK1/2. As shown in Figure 4C, xenograft tumor sections revealed signiﬁcantly increased p-ERK1/2 staining in shRASAL2-1-treated tumors compared with scramble-treated tumors. Moreover, we examined other core regulators of the ERK pathway in tumor samples via Western blotting. Higher levels of phospho-ERK1/2, phospho-MEK1/2 and phosphor-Raf-1 were consistently observed in RASAL2 knockdown tumor samples (Figure 4D). Taken together, these findings indicate that RASAL2 is an essential Ras-ERK pathway regulator in ovarian cancer.

### Ras-ERK pathway activation is required for RASAL2 suppression-mediated EMT and cell invasion in ovarian cancer

**Figure 5:**
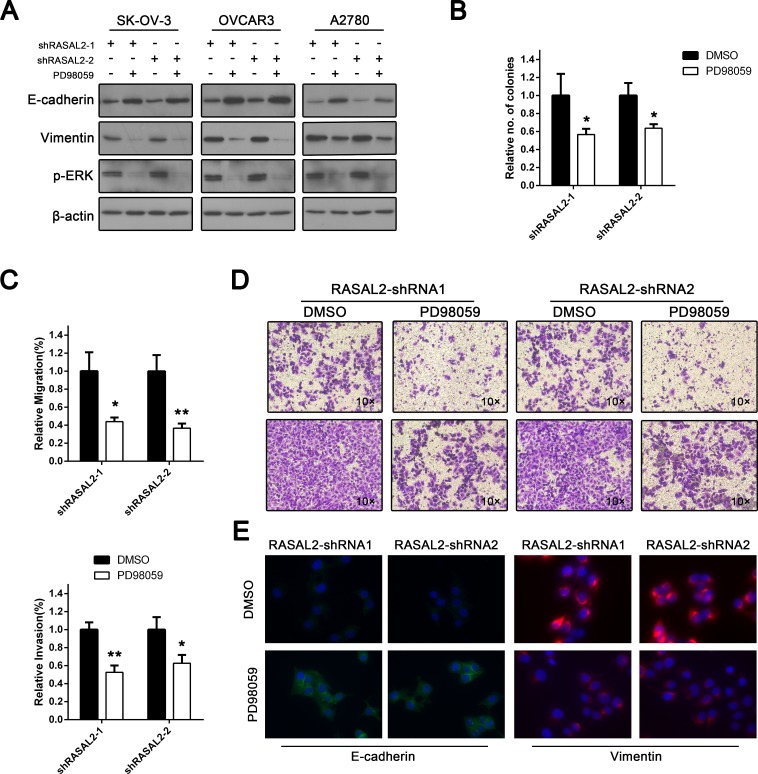
Tumor suppressor role of RASAL2 is Ras-ERK pathway-dependent To explore the functional effects of the Ras-ERK pathway following RASAL2 depletion, SK-OV-3, OVCAR3 and A2780 cells infected with shRASAL2-1 or -2 shRNA were treated with either the ERK pathway inhibitor PD98059 (10 μg/μl) or DMSO. (A) Western blot analysis of p-ERK, E-cadherin and vimentin expression following ERK inhibition. (B) SK-OV-3 cells expressing shRASAL2-1 or-2 shRNA were treated with PD98059 (10 μg/μl) or DMSO. Then, an anchorage-independent growth assay was performed. The graph indicates the relative number of colonies ± SD (n = 3). (C) Cell migration or invasion assays were also conducted on RASAL2 knockdown SK-OV-3 cells treated with either PD98059 or DMSO. All the data were normalized to the results of cells transfected with scramble-shRNA. The data are shown as the means ± SD (n = 3). (D) Representative graphs of SK-OV-3 cells in migration or invasion assays. (E) Immunofluorescence assay for the expression of E-cadherin or vimentin in RASAL2 knockdown SK-OV-3 cells treated with either PD98059 or DMSO. *, P < 0.05, * *, P < 0.01.

Because the Ras-ERK pathway plays an important role in EMT[22], we hypothesized that RASAL2 suppression-mediated EMT was ERK-dependent. To test this possibility, SK-OV-3, OVCAR3 and A2780 cells transfected with either shRASAL2-1 or shRASAL2-2 shRNA were incubated with PD98059, an ERK pathway inhibitor. Phospho-ERK down-regulation was confirmed by Western blot analysis (Figure 5A), and alterations in the expression of EMT-related proteins were examined. Immunoﬂuorescence analysis showed that ERK inhibition restored E-cadherin expression and localization to the cell-cell junctions (Figure 5E). Vimentin down-regulation was also confirmed via immunofluorescence. Moreover, expression alterations of E-cadherin and vimentin were also confirmed by Western blotting. These results were consistent with immunofluorescence analyses (Figure 5A).

Furthermore, we asked whether ERK inhibition had any impact on anchorage-independent growth of RASAL2-suppressed cells. As shown in Figure 5B, PD98059 significantly inhibited anchorage-independent colony growth of ovarian cancer cell lines transfected with RASAL2-shRNA1 or-2. Furthermore, both transwell migration and wound healing assays showed that RASAL2-depleted cells displayed suppressed migration following ERK inhibition (Figure 5C and 5D). The invasion of RASAL2 knockdown cells was also impaired (Figure 5C and 5D). Taken together, these results demonstrate that RASAL2 regulation of EMT and cell invasion is at least partially ERK-dependent.

## DISCUSSION

The lack of efficient early detection policies and limited treatments have led to a high mortality rate of ovarian cancer patients at advanced stages of disease[23]. Therefore, fully understanding the mechanisms of metastasis and searching for new targets have become prerequisites for improving therapies for ovarian cancer. In the current study, we found that the expression of RASAL2, a rarely reported GAP protein, was down-regulated during ovarian cancer. The reduction in the expression of RASAL2 paralleled the progression of FIGO stages and pathological grading, which indicates that low-expression of RASAL2 may relate to the progression of ovarian cancer. In addition, the depletion of RASAL2 promoted anchorage-independent growth of ovarian cancer cell lines (SK-OV-3, OVCAR3 and A2780) *in vitro*. Invasion and migration capabilities of ovarian cancer cell lines were significantly enhanced following RASAL2 suppression. Furthermore, RASAL2 depletion in SK-OV-3 cells significantly boosted tumor formation *in vivo*. Collectively, these results indicate that RASAL2 is a potential tumor suppressor gene, and down-regulation of its expression may play a role in promoting the progression of ovarian cancer. It was recently reported that RASAL2 functions as a tumor suppressor in breast cancer[17]. Down-regulation of RASAL2 expression is observed in primary human breast cancer and is associated with recurrence and metastasis. These results are consistent with our report and imply that the regulatory effect of RASAL2 on tumor progression may exist for a broad spectrum of malignant diseases.

Unlike most solid tumors, ovarian cancer spreads mainly via implantation within the peritoneal cavity, while hematogenous metastasis is seldom observed[19, 24, 25]. Invasion and migration capacity of ovarian cancer cells plays a key role in the metastasis process. Epithelial-mesenchymal-transition (EMT) enables tumor cells to gain invasion and migration capabilities, and this phenomenon has been reported for various cancers, such as skin, head and neck, breast, lung, liver, colon and prostate cancer. Recently, several studies reported the role of EMT in ovarian cancer malignancy characteristics[23, 26-29]. However, the mechanisms of EMT regulation by ovarian cancer are still unclear. In the current study, we found that the low expression of RASAL2 *in vitro* could significantly promote the EMT process, including up-regulation of the expression of mesenchymal-related markers, such as vimentin and N-cadherin, and down-regulation of the expression of epithelial phenotype-related markers, such as E-cadherin, claudin-1 and ZO-1, in ovarian cancer cell lines. In addition, RASAL2 suppression could up-regulate the expression of EMT transcription factors, including Snail and Slug. Accordant results were also observed after RASAL2 suppression in an *in vivo* xenograft model. Furthermore, RASAL2 expression was negatively correlated with E-cadherin in primary human ovarian cancer. In conclusion, we have identified RASAL2 as an EMT regulatory protein in ovarian cancer. This study not only provides evidence of EMT in ovarian cancer but also suggests a novel mechanism by which ovarian cancer regulates the EMT process.

Activation of the Ras pathway is commonly observed in ovarian cancer, especially in type I ovarian cancer, in which the activation rate exceeds 60%[30]. Several studies have confirmed that activation of the Ras pathway promotes the progression of ovarian cancer. Ras, together with its two regulatory proteins, guanine nucleotide exchange factors (GEFs) and GTPase-activating proteins (GAPs), serve as a switch for signaling pathways[14, 15]. However, most studies have attributed the activation of Ras pathway to *Ras* mutations[31-33], while few reports have examined alterations in post-translational modifications of the Ras protein. In the current study, we found that the Ras-ERK pathway was activated both *in vivo* and *in vitro* following RASAL2 suppression. Furthermore, blockade of the Ras-ERK pathway using inhibitors weakened invasion, migration, anchorage-independent growth and EMT. These results confirm the existence of post-translational regulation of Ras activation in ovarian cancer, which is mediated by RASAL2. Importantly, this mechanism affects the progression of ovarian cancer.

Our study shows that the expression of RASAL2 is negatively correlated to FIGO stages and pathological grading, which indicates that low expression of RASAL2 may relate to the progression of ovarian cancer. RASAL2 also participates in the regulation of cancer cell invasion and migration. Hence, RASAL2 may be a potential biomarker of clinical staging and grading and may possibly be a biomarker for prognosis assessment. However, the application value of clinical examination still needs further evaluation. As the Ras-ERK pathway is activated in a wide range of cancers and functions as a promoting factor, many researchers have focused on this pathway and have searched for targeted therapies. However, the results have not been satisfactory until now mainly due to the complexity of the Ras-ERK pathway and its rich compensatory mechanism[34, 35]. Our research shows that activation of the Ras pathway can be attributed to mechanisms other than mutation, which may suggest novel strategies to target the Ras pathway. Because down-regulation of RASAL2 occurs during ovarian cancer, restoring RASAL2 expression or synthesizing a small molecule that can replace RASAL2 and recover the hydrolysis of GTP by Ras may become a novel approach to cancer therapies.

Intensive efforts were made to discover new therapeutic targets and inhibitors to treat cancer[36-38]. Numerous agents targeting growth factor receptors, mTOR, PI-3K, and related tyrosine kinases, Ras, Raf, and B-Raf, S6K, MEK1/2 have been tested. Among which, MEK and Raf inhibitors received much concern. MEK inhibitors in clinical development or undergo clinical trials include selumetinib, pimasertib, refametinib, PD-0325901, TAK733, MEK162, RO5126766, WX-554, RO4987655, GDC-0973, and AZD8330. Trametinib, an MEK inhibitor, has been utilized in clinical managements of malignant diseases[39, 40]. Studies on pathway-inhibitor treatment in ovarian cancer also showed great significance[36, 38]. In the current study, PD98059, also a MEK inhibitor, efficiently attenuated the intensity in the invasive and migration, anchorage-independent growth and EMT induced by RASAL2 suppression in ovarian cancer cells. Although PD98059 was shown to be an effective anti-tumor agent in this study, whether other MEK inhibitors have similar effects need further evaluation.

Ras activates not only the MAPK pathway, but also PI3K-AKT pathway. Recent report indicates that MEK can also activate mTOR pathway[41]. Therefore the PI3K-AKT pathway may also participate in EMT which was mediated by down-regulation of RASAL2, raising the possibility that the PI3K-AKT pathway inhibitors or mTOR pathway inhibitors, such as rapamycin, may also be effective in the treatment of Ras pathway-activated ovarian cancer. Further studies is needed to ascertain this.

In summary, we show that RASAL2, which is down-regulated in ovarian cancer, is a novel suppressor of EMT and metastasis, and its regulation of EMT and metastasis relies on activation of the Ras-ERK pathway. These results provide a baseline for further exploration into EMT and metastasis in ovarian cancer. We propose RASAL2 as a potential therapeutic target and prognostic marker for ovarian cancer; however, further evaluation is still required. Whether other proteins in the RasGEF and RasGAP families also participate in the progression of ovarian cancer is another question to be resolved. We believe that uncovering these problems will provide key information towards our understanding of the occurrence and development of ovarian cancer and its treatment.

## MATERIALS AND METHODS

### Cell lines and reagents

Ovarian cancer cell lines (SK-OV-3, OVCAR3 and A2780) were purchased from Type Culture Collection of Chinese Academy of Sciences and maintained according to their recommendations. The ERK inhibitor PD98059 was obtained from Sigma. Antibodies were obtained from the following companies: anti-RASAL2 antibody from Abcam; anti-β-actin antibody from Sigma; anti-E-cadherin, anti-vimentin, anti-claudin-1, anti-N-cadherin, anti-ZO-1, anti-Snail, anti-Slug, anti-Raf-1, anti-p-Raf-1, anti-MEK1/2, anti-p-MEK1/2, anti-ERK1/2 and anti-p-ERK1/2 antibodies from Cell Signaling.

### Ras Activation Assay

Ras activation was measured by a Ras Activation ELISA Assay Kit (Millipore) following the manufacturer's instruction. Briefly, wells were incubated with Raf-1-RBD at 4°C for 1 hour, and then the cell lines and tissue samples were added and incubated at room temperature for 1 hour. Primary antibody solution and secondary antibody solution were each incubated at room temperature for 1 hour, and then a chemiluminescent substrate was added. The results were read using a GloMax^®^ 20/20 luminometer 30 minutes after adding the substrate.

### Anchorage-independent growth assay

Cells were plated in triplicate in 6-well dishes using the appropriate growth media for each cell line and low melting point agarose (Sigma). Plating conditions were as follows: SK-OV-3: 10,000 cells, 0.6% agar base, 0.35% agar top; OVCAR3 cells: 5,000 cells, 0.6% agar base, 0.35% agar top; A2780: 10,000 cells, 0.6% agar base, 0.35% agar top. Colonies were counted from 5 representative fields per well after 2-4 weeks.

### Lentiviral infections

RASAL2-shRNA and control lentivirus were obtained from Shanghai Genepharma Co., Ltd. The RASAL2-shRNA1 target sequence was 5′-CCCTCGTGTTCTTGCTGATAT-3′. The RASAL2-shRNA2 target sequence was 5′-GCCTTCCACCTCTTCATAGTA-3′. Virus supernatant was incubated on target cells for 12 hours with 8 μg/ml polybrene, following the manufacturer's instructions. Infected cells were selected in puromycin, as optimized for each cell line.

### Protein extraction and Western blot analysis

Whole-cell protein was isolated as described previously[42]. For tissue protein extraction, the tumor tissues were homogenized in ice-cold RIPA (radioimmunoprecipitation assay) lysis buffer (Millipore). The tissue lysates were incubated at 4°C for 1 hour with rotation, followed by clariﬁcation of tissue debris by centrifugation at 12,000 rpm for 10 minutes. The protein concentration of tumor extracts was determined using the BCA Protein Assay Kit (Pierce). Western blotting were performed as previously described[42]. Antibody binding was revealed using an HRP-conjugated anti-rabbit IgG or anti-mouse IgG (Sigma). Antibody complexes were detected using Immobilon Western Chemiluminescent HRP Substrate (Millopore) and exposure to X-Omat ﬁlm (Kodak).

### Immunohistochemistry

Murine tumors were ﬁxed in 10% neutral buffered formalin for 24 hours. Then, tumors were processed for parafﬁn embedding. Of note, 5 mmol/L sections were used for hematoxylin and eosin staining and immunohistochemistry. Unstained sections were deparafﬁnized, rehydrated and stained for RASAL2 (1:400; Abcam), p-ERK (1:400; Cell Signaling Technology) and E-cadherin (1:400; BD Biosciences).

### Immunofluorescence analysis

Cells were grown on glass coverslips, fixed with 4% paraformaldehyde for 15 min, and blocked with phosphate-buffered saline (PBS) containing 5% fetal bovine serum for 60 min. Cells were incubated with primary antibody in PBS at 4°C overnight, washed three times with PBS, incubated with Alexa Fluor 488 or Alexa Fluor 546-labeled secondary antibody (Invitrogen) in PBS for 1 h, and then analyzed using a confocal fluorescence microscope (FV1000-D, Olympus, Tokyo, Japan).

### Migration and Invasion Assays

For the transwell migration assay, ovarian cancer cells were trypsinized and placed in the upper chamber of each insert (Corning, Cambridge, USA) containing the non-coated membrane. For the invasion assay, cells were placed on the upper chamber of each insert coated with 40 μl of matrigel (BD biosciences), which was diluted to 4 μg/μl with serum-free medium. Then, medium supplemented with 20% fetal bovine serum (600 μl) was added to the lower chambers. After 24 hours of incubation at 37°C, the upper surface of the membrane was wiped with a cotton tip, and the cells attached to the lower surface were stained for 10 min with crystal violet. Cells in five random fields of view at ×100 magnification were counted and expressed as the average number of cells per field of view. All assays were performed in triplicate.

### Wound healing assay

Ovarian cancer cells were 95-100% confluent. Wounds were generated with a pipette tip, and images of wound healing (migration) were taken at the indicated time intervals.

### Apoptosis and proliferation assay

Apoptosis rate and proliferation rate were measured by a PE Annexin V Apoptosis Detection Kit (BD Pharmingen) and a Cell Proliferation ELISA Kit (Roche) respectively. The measurements were performed following the manufacture's instruction.

### RNA isolation and qRT-PCR

Total RNA was isolated using Trizol reagent (Invitrogen, Carlsbad, USA). Total RNA (2 μg) was used for the synthesis of first-strand cDNA using M-MLV reverse transcriptase (Invitrogen, Beijing, China). Quantitative real-time PCR was performed using the SYBR green mix (Applied Biosystems). The reactions were performed with a 7500 Fast Real-Time PCR System (Applied Biosystems). The data were displayed as 2-ΔΔCt values and were representative of at least three independent experiments. Sequences of the RT-PCR primers were as follows (5′- 3′):

RASAL2

(AGCAGAAAGGTCCCCTCGTAG and AGGGTGAGGTATTTGCAGTGT)

GAPDH

(GGAGCGAGATCCCTCCAAAAT and GGCTGTTGTCATACTTCTCATGG)

E-cadherin

(CGAGAGCTACACGTTCACGG and GGGTGTCGAGGGAAAAATAGG)

Vimentin

(GACGCCATCAACACCGAGTT and CTTTGTCGTTGGTTAGCTGGT)

Claudin-1

(CCTCCTGGGAGTGATAGCAAT and GGCAACTAAAATAGCCAGACCT)

N-cadherin

(TCAGGCGTCTGTAGAGGCTT and ATGCACATCCTTCGATAAGACTG)

ZO-1

(CAACATACAGTGACGCTTCACA and CACTATTGACGTTTCCCCACTC)

SNAIL

(TCGGAAGCCTAACTACAGCGA and AGATGAGCATTGGCAGCGAG)

Slug

(CGAACTGGACACACATACAGTG and CTGAGGATCTCTGGTTGTGGT)

### Ovarian cancer xenograft models

Female athymic nude mice were purchased from Academy of Military Medical Science (Beijing, China). All mouse studies were approved by the Animal Ethics Committee of Tianjin Medical University. All animals were 4-6 weeks of age at the time of injection. SK-OV-3-scramble and SK-OV-3-shRASAL2-1 cells were trypsinized, washed, resuspended in Hanks′ balanced salt solution (GIBCO) and injected into the peritoneal cavities of mice (2×10^6^ cells/animal).

### Statistical Analysis

Data are reported as mean values ± SEM. Biochemical experiments were performed in triplicate and a minimum of three independent experiments were evaluated. Differences were assessed for statistical signiﬁcance by an unpaired two-tailed t test, by the log rank test (for Kaplan-Meier plots). The p values are considered as follows: *p < 0.05; **p < 0.01.

## SUPPLEMENTARY MATERIAL FIGURES AND TABLE


