# Supplementary material for: RASAL2 down-regulation in ovarian cancer promotes epithelial-mesenchymal transition and metastasis
Source: Oncotarget. 2014 Jul 23;5(16):6734–45. doi: 10.18632/oncotarget.2244 (PMC4196159; doi:10.18632/oncotarget.2244)
Supplement: Supplementary file 1 [file oncotarget-05-6734-s001.pdf]

RASAL2 down-regulation in ovarian cancer promotes epithelial-mesenchymal transition and metastasis

Supplementary Material

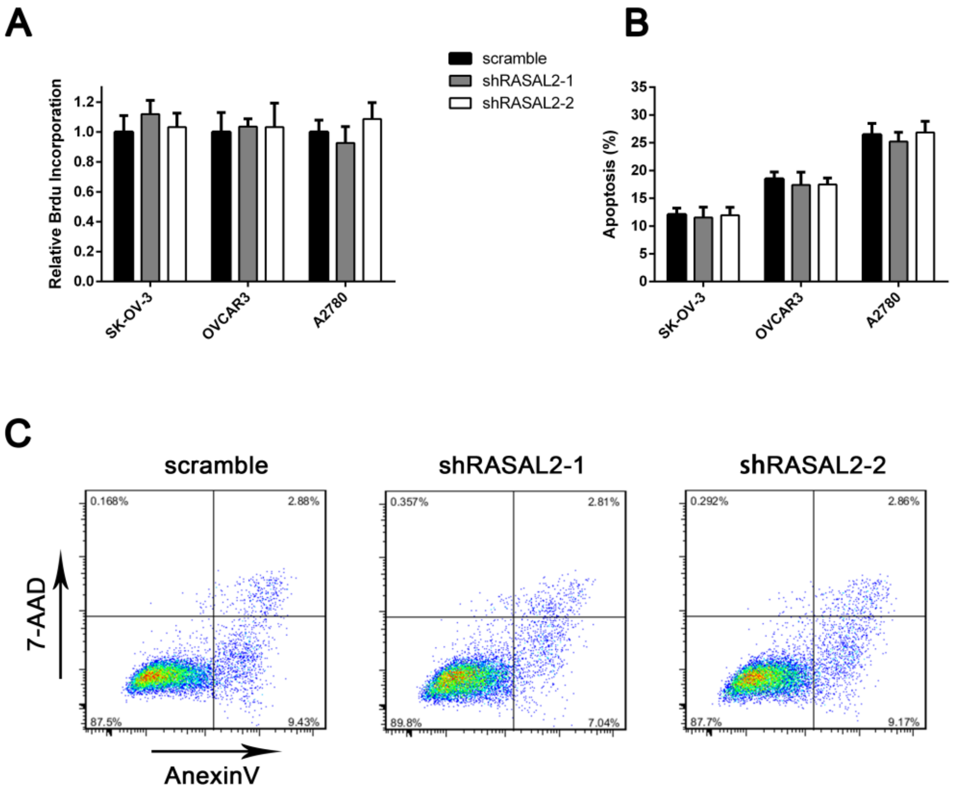

Supplementary Figure 1: RASAL2 depletion does not affect ovarian cancer cell proliferation and cisplatin induced apoptosis (A) Assay for cell proliferation on SK-OV-3, OVCAR3 and A2780 cells infected with shRASAL2-1, shRASAL2-2 or scramble-shRNA by a Cell Proliferation ELISA (BrdU) kit. All the data were normalized to the results of cells transfected with scramble-shRNA. The data are shown as the means ± SD (n=3). (B) SK-OV-3, OVCAR3 and A2780 cells infected with shRASAL2-1, shRASAL2-2 or scramble-shRNA were treated with cisplatin (10μg/ml) for 24h, then examined for apoptosis by flow cytometry. The data are shown as the means ± SD (n=3). (C) Representative graphs of SK-OV-3 cells apoptosis results. *, $P < 0.05$, * *, $P < 0.01$.

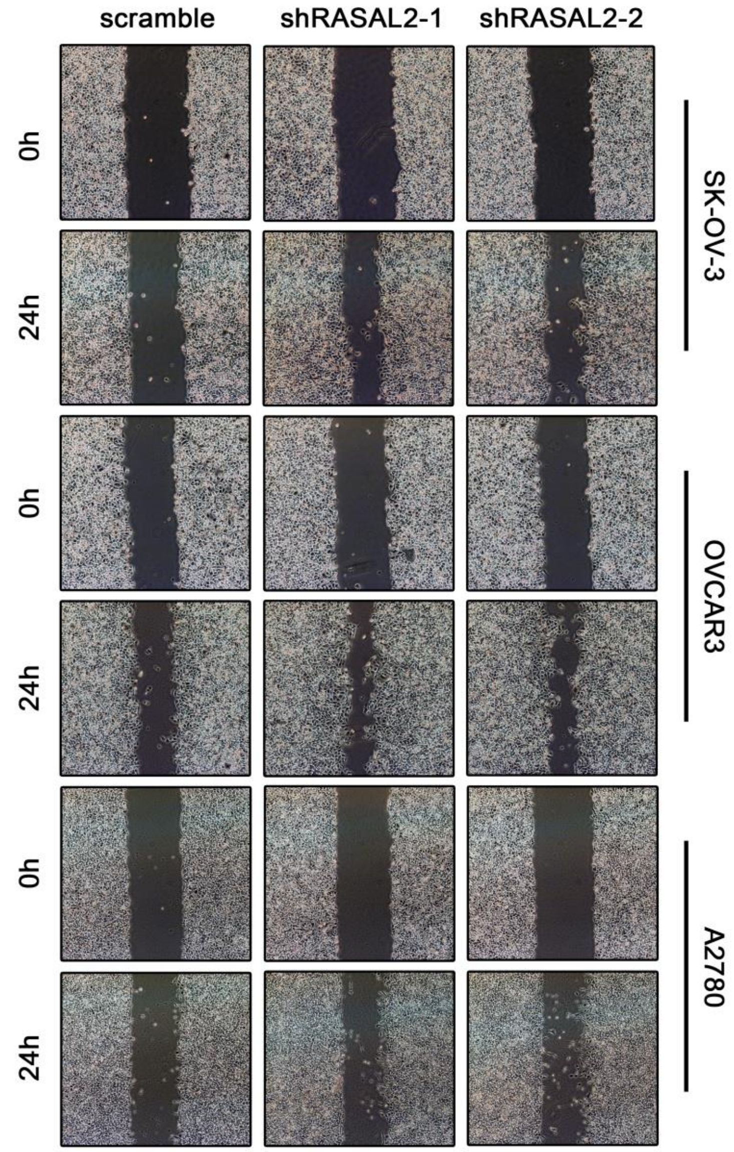

Supplementary Figure 2: RASAL2 depletion enhanced ovarian cancer cells migration SK-OV-3, OVCAR3 and A2780 cells infected with shRASAL2-1, shRASAL2-2 or scramble-shRNA were subjected for wound healing assay.

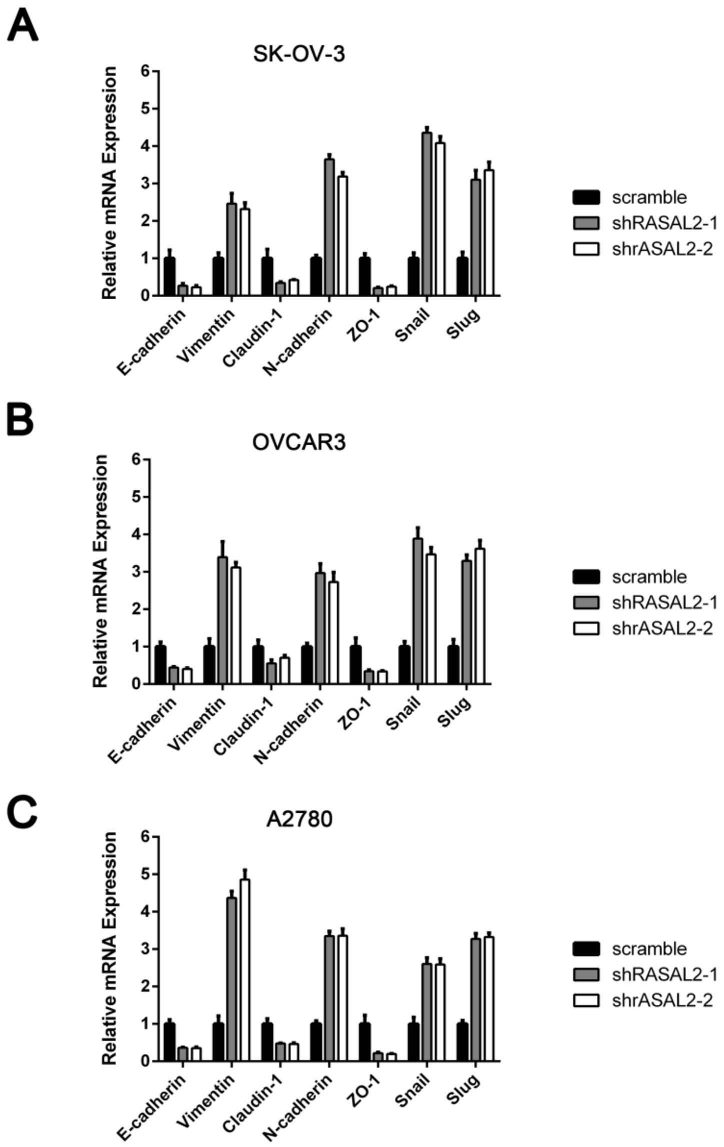

Supplementary Figure 3: RASAL2 depletion altered mRNA expression of EMT markers (A, B and C) qPCR analysis of EMT markers levels in SK-OV-3, OVCAR3 and A2780 cells infected with shRASAL2-1, shRASAL2-2 or scramble-shRNA. All the data were normalized to the results of cells transfected with scramble-shRNA. The data are shown as the means ± SD (n=3). *, P < 0.05, * *, P < 0.01.

Supplementary Table-1:

Details of patients' samples

study_id	Age	Tumor_stage	Tumor_grade	Histology	Epithelial_type
OC_01	20	I	Grade1	epithelial	mucous
OC_02	48	II	Grade1	epithelial	mucous
OC_03	14	I	Grade1	epithelial	mucous
OC_04	56	IV	Grade2	epithelial	serous
OC_05	53	II	Grade3	epithelial	serous
OC_06	50	II	Grade3	epithelial	serous
OC_07	47	II	Grade3	epithelial	serous
OC_08	39	III	Grade1	epithelial	serous
OC_09	35	IV	Grade1	epithelial	serous
OC_10	60	IV	Grade2	epithelial	serous
OC_11	61	II	Grade2	epithelial	serous
OC_12	65	II	Grade2	epithelial	mucous
OC_13	38	I	Grade1	epithelial	serous
OC_14	67	II	Grade2	epithelial	serous
OC_15	59	I	Grade1	epithelial	serous
OC_16	58	II	Grade3	epithelial	endometrioid
OC_17	60	II	Grade3	epithelial	endometrioid
OC_18	51	II	Grade3	epithelial	endometrioid
OC_19	44	IV	Grade3	epithelial	endometrioid
OC_20	64	IV	Grade1	epithelial	serous
OC_21	58	IV	Grade2	epithelial	mucous
OC_22	54	II	Grade3	epithelial	clear cell carcinoma
OC_23	51	IV	Grade2	epithelial	serous
OC_24	73	IV	Grade3	epithelial	serous
OC_25	57	IV	Grade2	epithelial	serous
OC_26	56	IV	Grade2	epithelial	serous
OC_27	61	IV	Grade3	epithelial	endometrioid
OC_28	53	III	Grade3	epithelial	serous
OC_29	64	III	Grade3	epithelial	clear cell carcinoma
OC_30	44	IV	Grade3	epithelial	serous
OC_31	53	IV	Grade3	epithelial	endometrioid
OC_32	72	IV	Grade2	epithelial	serous
OC_33	50	III	Grade3	epithelial	endometrioid
OC_34	58	III	Grade3	epithelial	endometrioid
OC_35	64	IV	Grade3	epithelial	serous
OC_36	45	III	Grade2	epithelial	endometrioid

OC_37	60	IV	Grade3	epithelial	endometrioid
OC_38	50	IV	Grade3	epithelial	mucous
OC_39	61	III	Grade3	epithelial	clear cell carcinoma
OC_40	42	IV	Grade3	epithelial	endometrioid
OC_41	66	II	Grade3	epithelial	mucous
OC_42	51	III	Grade2	epithelial	serous
OC_43	47	IV	Grade3	epithelial	mucous
OC_44	68	IV	Grade3	epithelial	serous
OC_45	59	IV	Grade2	epithelial	serous
OC_46	69	IV	Grade3	epithelial	serous
OC_47	69	IV	Grade2	epithelial	serous
OC_48	45	II	Grade3	epithelial	endometrioid
OC_49	74	III	Grade3	epithelial	endometrioid
OC_50	56	IV	Grade2	epithelial	serous
OC_51	69	II	Grade3	epithelial	clear cell carcinoma
OC_52	72	IV	Grade2	epithelial	serous
OC_53	68	IV	Grade3	epithelial	endometrioid
OC_54	41	II	Grade3	epithelial	serous
OC_55	67	III	Grade3	epithelial	mucous
OC_56	44	IV	Grade3	epithelial	serous
OC_57	48	IV	Grade3	epithelial	serous
OC_58	38	N	N	epithelial	NA
OC_59	56	N	N	epithelial	NA
OC_60	44	N	N	epithelial	NA
OC_61	48	N	N	epithelial	NA
OC_62	39	N	N	epithelial	NA
OC_63	59	N	N	epithelial	NA
OC_64	61	N	N	epithelial	NA
OC_65	62	N	N	epithelial	NA
